# Pathological nociceptors in two patients with erythromelalgia‐like symptoms and rare genetic Nav 1.9 variants

**DOI:** 10.1002/brb3.528

**Published:** 2016-07-21

**Authors:** Inge P. Kleggetveit, Roland Schmidt, Barbara Namer, Hugh Salter, Tormod Helås, Martin Schmelz, Ellen Jørum

**Affiliations:** ^1^ Section of Clinical NeurophysiologyDepartment of NeurologyOslo University Hospital‐RikshospitaletOsloNorway; ^2^ Department of Clinical NeurophysiologyUppsala UniversityUppsalaSweden; ^3^ Institute of Physiology and PathophysiologyFriedrich‐Alexander‐Universität Erlangen‐NürnbergErlangenGermany; ^4^ AstraZeneca Translational Science CentreDepartment of Clinical NeuroscienceKarolinska InstitutetSolnaSweden; ^5^ Department of Anesthesiology MannheimHeidelberg UniversityMannheimGermany

**Keywords:** microneurography, nociceptors, pain

## Abstract

**Introduction:**

The sodium channel Nav 1.9 is expressed in peripheral nociceptors and has recently been linked to human pain conditions, but the exact role of Nav 1.9 for human nociceptor excitability is still unclear.

**Methods:**

C‐nociceptors from two patients with late onset of erythromelalgia‐like pain, signs of small fiber neuropathy, and rare genetic variants of Nav 1.9 (N1169S, I1293V) were assessed by microneurography.

**Results:**

Compared with patients with comparable pain phenotypes (erythromelalgia‐like pain without Nav‐mutations and painful polyneuropathy), there was a tendency toward more activity‐dependent slowing of conduction velocity in mechanoinsensitive C‐nociceptors. Hyperexcitability to heating and electrical stimulation were seen in some nociceptors, and other unspecific signs of increased excitability, including spontaneous activity and mechanical sensitization, were also observed.

**Conclusions:**

Although the functional roles of these genetic variants are still unknown, the microneurography findings may be compatible with increased C‐nociceptor excitability based on increased Nav 1.9 function.

## 1 Introduction

The sodium channel isoforms Nav 1.7, Nav 1.8, and Nav 1.9 are preferentially expressed in peripheral neurons (Dib‐hajj, Cummins, Black, & Waxman, 2010) and may be of special importance for human nociception and pain. Erythromelalgia is a painful condition characterized by pain in red/hot distal extremities typically relieved by cooling and worsened by warm temperatures (Kurzrock & Cohen, 1991), which may be associated with peripheral neuropathy (Orstavik, Mork, Kvernebo, & Jorum, 2004). Interestingly, mutations of Nav 1.7 are closely linked to primary erythromelalgia (Dib‐hajj et al., 2010) but also to other painful conditions including small fiber neuropathy (Faber, Hoeijmakers, et al., 2012). In addition, mutations of Nav 1.8 have been found in patients with painful neuropathy (Faber, Lauria, et al., 2012) and recently, mutations of Nav 1.9 were not only linked to both episodic pain (Zhang et al., 2013), cold‐aggravated pain (Leipold et al., 2015), and painful neuropathy (Han et al., 2015 *;* Huang et al., 2014) but also to pain insensitivity (Leipold et al., 2013). Normally, these different sodium channel isoforms probably serve different aspects important for generation and conduction of nerve signals in subgroups of peripheral neurons. While Nav 1.7 may be important by providing ramp currents (acting as a “threshold channel”) and Nav 1.8 has been suggested to be responsible for the “up‐stroke” of action potentials, Nav 1.9 may produce a persistent sodium current and increase the response to subthreshold depolarizations (Dib‐hajj et al., 2010). However, the exact role of Nav 1.9 in human nociceptor excitability is problematic to evaluate due to difficulties with recording from small nociceptive endings (Vasylyev & Waxman, 2012), differences between species (Dib‐hajj, 2014), and difficulties in heterologous expression of Nav 1.9 (Gilchrist & Bosmans, 2012). This adds to the more general problems regarding translation of results from preclinical research (e.g., species differences, relevance of pain models) into clinical medicine, a challenge which is particularly relevant for the field of pain. The method of microneurography may in this respect provide unique information, since it allows direct recordings of action potentials from single nerve fibers in humans (Namer & Handwerker, 2009). Here, we assessed microneurography recordings from C‐nociceptors in two patients with a late debut of erythromelalgia‐like symptoms, signs of small fiber neuropathy, and rare genetic variants of Nav 1.9.

## 2 Materials and Methods

The two adult female patients underwent assessment of thermal detection thresholds (as described elsewhere (Warncke, Jorum, & Stubhaug, 1997), electromyography/neurography as well as microneurography recordings from individual cutaneous C‐fibers (including C‐nociceptors) in the peroneal nerve (Kleggetveit et al., 2012; Orstavik et al., 2003; Schmelz et al., 1995). Based on their sensory and axonal characteristics assessed by microneurography, C‐nociceptors were further subclassified as either mechanosensitive (CM‐) or mechanoinsensitive (CMi‐) nociceptors. C‐nociceptors which were not possible to subclassify into one of these subgroups, were denominated C‐nociceptors of unknown type (C‐nou). In an attempt to identify specific nociceptor changes for these two patients, the microneurography recordings were compared to control data from previous recordings from patients with comparable pain phenotypes (erythromelalgia‐like symptoms without Nav channel mutations and peripheral neuropathic pain). The microneurography recordings were performed in several sessions. For patient 1, three recording sessions were performed, and parts of data from the two initial sessions have been published previously in another context (Kleggetveit et al., 2012; Schmidt et al., 2012). We also add data from a later third recording session and present an extended analysis of the data, given the information that patient 1 was identified with a rare genetic variant of Nav 1.9 (Zhang et al., 2014). Data from the two recording sessions from patient 2 have not been previously presented. Parts of the (historical) control data (microneurography recordings from patients with erythromelalgia without similar mutations and patients with peripheral neuropathic pain [without known mutations]) have been published previously (Kleggetveit et al., 2012; Namer et al., 2015; Schmidt et al., 2012). The genetic variants of Nav 1.9 were described for patient 1 (N1169S) and patient 2 (I1293V) in a separate study (Zhang et al., 2014). The study was approved by the local ethical committee and the patients provided an informed consent.

## 3 Results

The patients reported symptoms starting at the age of 57 and 49 with ongoing pain of predominantly burning character and red/warm hands and feet, typically being relieved by cooling. They did not report distinct pain attacks. Electromyography/neurography showed no clear signs of large fiber pathology, while thermal detection thresholds from the feet dorsum indicated small fiber neuropathy without signs of heat hyperalgesia: 26.8°C/46.8°C/>50°C (cold/warmth/heat pain, respectively) for patient 1 and 25.1°C/43.4°C/46.7°C for patient 2.

A summary of microneurography results are listed in Table 1. Axonal characteristics of the C‐fibers in the two patients did not in general differ from other EM patients, but a tendency toward more activity‐dependent slowing of conduction velocity (ADS) in their CMi‐nociceptors was observed exceeding the level from controls (patients with comparable pain phenotypes) by more than 50% (ADS 1/8 (%)). Moreover, for some fibers, we tested velocity changes induced by paired stimuli (Weidner et al., 2000; interstimulus intervals 15–2,000 ms; “velocity recovery cycles”) which provided a similar pattern when compared to the control patients: in the two patients, supranormal conduction was more pronounced in CMi‐nociceptors, whereas CM‐nociceptors (data only from patient 1) did not differ from control EM patients (data only shown for interstimulus intervals 40 and 15 ms). When testing heat activation thresholds of individual C‐nociceptors, low (i.e., below 40.0°C) heat activation thresholds in four CM‐nociceptors and five C‐nociceptors of unknown type were found in patient 1, while in patient 2, a low heat threshold in one CM‐nociceptor was observed. Also, other signs of nociceptor hyperexcitability relevant for pain such as spontaneous activity, mechanical sensitization (of CMi‐nociceptors), and multiple spikes (Schmidt et al., 2012) were observed.

**Table 1:** Microneurography data from C‐nociceptors and C‐sympathetic fibers in two patients with genetic variants of Nav 1.9 and patients with comparable pain conditions

	Patient 1 (1169S)	Patient 2 (I1293V)	EM	NP
Spont.act.				
CM	3 (10)	0 (2)	5 (23)	4 (39)
Cmi	1 (8)	1 (6)	4 (8)	12 (43)
C‐nou	6 (10)	0 (1)	10 (14)	2 (12)
Mech resp				
CM	10 (10)	2 (2)	23 (23)	39 (39)
Cmi	2 (7)	3 (6)	0 (8)	12 (41)
C‐nou	6 (6)	1 (1)	7 (14)	10 (11)
Heat resp				
CM	5 (5)	1 (1)	16 (17)	29 (32)
Cmi	2 (6)	1 (1)	5 (5)	17 (33)
C‐nou	5 (5)	1 (1)	8 (11)	8 (10)
Heat th (°C)				
CM	38.0 (37.0, 40.0) (5)	38.0 (1)	42.5 (37.8, 44.3) (14)	43.0 (41.0, 44.5) (21)
Cmi	45.5 (45.0, 46.0) (2)	42.5 (1)	43.0 (42.0, 43.0) (3)	42.0 (41.0, 47.0) (15)
C‐nou	38.0 (37.5, 39.9) (5)	<42.7 (1)	46.2 (43.6, 49.4) (8)	46.0 (41.0, 46.5) (5)
CV (m s^−1^)				
CM	0.98 (0.94, 1.04) (10)	1.16 (1.15, 1.17) (2)	1.00 (0.95, 1.04) (22)	0.97 (0.83, 1.05) (39)
Cmi	0.75 (0.58, 0.95) (8)	0.82 (0.75, 0.90) (6)	0.61 (0.51, 0.73) (8)	0.71 (0.61, 0.82) (43)
C‐nou	0.65 (0.57, 0.98) (10)	0.93 (1)	0.88 (0.74, 0.94) (14)	0.84 (1.01, 0.71) (12)
Symp	0.72 (0.66, 0.84) (32)	0.94 (0.70, 0.99) (4)	0.86 (0.65, 0.95) (10)	0.80 (0.73, 0.87) (26)
ADS1/8 (%)				
CM	0.12 (−0.30, 0.34) (10)	0.35 (0.23, 0.46) (2)	0.18 (−0.02, 0.33) (22)	0.12 (0.01, 0.25) (38)
Cmi	2.13 (1.45, 2.56) (5)	2.01 (1.22, 2.33) (6)	1.30 (1.06, 1.59) (8)	1.29 (0.83, 1.70) (33)
C‐nou	0.14 (0.08, 0.20) (2)	n.a	0.39 (0.19, 0.63) (13)	0.23 (−0.06, 0.62) (10)
Symp	0.59 (0.22, 0.80) (15)	n.a	0.60 (0.44, 0.70) (10)	0.38 (0.26, 0.55) (9)
ADStot (%)				
CM	1.66 (0.93, 2.50) (9)	2.78 (2.36, 3.21) (2)	1.74 (1.02, 2.32) (22)	2.13 (1.71, 2.41) (37)
Cmi	8.18 (7.06, 10.05) (4)	9.16 (6.86, 11.11) (6)	6.70 (6.22, 7.10) (8)	7.59 (6.43, 8.94) (31)
C‐nou	4.39 (1)	n.a	3.80 (3.55, 4.63) (14)	4.78 (4.07, 5.75) (10)
Symp	3.60 (3.06, 4.64) (15)	n.a	2.86 (2.39, 3.35) (10)	3.19 (2.43, 4.07) (10)
Rec10 (%)				
CM	49.0 (37.0, 53.5) (9)	49.0 (37.2, 60.9) (2)	40.4 (31.8, 56.5) (22)	51.2 (46.2, 60.6) (37)
Cmi	18.8 (16.8, 23.2) (4)	10.6 (9.3, 12.6) (6)	13.0 (11.2, 17.0) (8)	20.5 (13.3, 22.7) (31)
C‐nou	47.5 (1)	n.a	14.6 (9.6, 18.6) (9)	46.9 (38.4, 53.9) (10)
Symp	26.0 (21.8, 28.2) (15)	n.a	23.5 (14.5, 26.5) (9)	30.6 (28.7, 38.2) (9)
ISI 40				
CM	3.1 (2.6, 3.5) (2)	n.a.	3.1 (3.1, 3.2) (10)	2.9 (1.9, 4.5) (14)
Cmi	−2.3 (−2.9, −1.9) (5)	−1.2 (−1.9, −0.5) (2)	0.9 (−0.1, 2.0) (3)	−0.2 (−0.9, 0.6) (17)
C‐nou	2.6 (1)	n.a.	1.2 (0.4, 3.2) (3)	2.0 (1.2, 2.2) (4)
Symp	−1.8 (−2.7, −1.5) (5)	n.a.	0.3 (−0.3, 0,9) (2)	−2.8 (1)
ISI 15				
CM	4.5 (3.9, 5.1) (2)	n.a.	4.1 (3.5, 4.8) (9)	4.5 (4.2, 4.9) (13)
Cmi	−0.5 (−0.8, 0.1) (4)	0.6 (0.4, 0.8) (2)	3.9 (3.5, 4.4) (2)	1.4 (0.3, 2.1) (12)
C‐nou	4.3 (1)	n.a.	4.0 (3.0, 5.0) (3)	4.0 (3.4, 6.8) (3)
Symp	0.3 (0.0, 0.4) (5)	n.a.	3.0 (2.3, 3.8) (2)	−0.1 (1)

EM, Patients with erythromelalgia (without Nav mutations); NP, Patients with peripheral neuropathic pain; CM, C‐mechanosensitive nociceptors; Cmi, C‐mechanoinsensitive nociceptors; C‐nou, C‐nociceptors of unknown type; Symp, Sympathetic C‐fibers; n.a., Not available; Sp. act, Spontaneous activity; Mech pos, Positive response to mechanical stimulation (von Frey 750 mN); Heat pos, Positive response to heat ramp (32–50°C, rate 0.25°C s^−1^). Numbers for sp. act, mech. pos, and heat. pos are given as *n* = positive fibers (*n* = tested fibers); Heat th, heat threshold in heat ramp; CV, Conduction velocity; ADS1/8, activity‐dependent slowing after 20 electrical pulses at 0.125 Hz (in % of CV); ADStot: Total activity‐dependent slowing after a low frequency protocol (20 pulses at 0.125 Hz, 20 pulses at 0.25 Hz, and 30 pulses at 0.5 Hz; in % of CV). Rec 10, Recovery of conduction velocity at the 10th pulse after the low frequency protocol; ISI 40 and ISI 15: To test for short‐term excitability changes (velocity recovery cycles), conduction velocity slowing (positive values), or speeding (negative values) of a trailing action potential was assessed as a function of time interval (interstimulus intervals at 40 and 15 ms are shown) between leading and trailing action potential. Numbers for CV, ADS1/8, ADStot, Rec10, and ISI are given as median (25th, 75th percentiles) (*n* = tested fibers), but range is given instead of percentiles when *n *<* *4. Values are given in percentage of conduction velocity. Parts of the data from patient 1 and control patients (EM and NP) have been published previously in other contexts (Kleggetveit et al., 2012 *;* Namer et al., 2015 *;* Schmidt et al., 2012 *;* see Materials and Methods).

**Figure 1:**
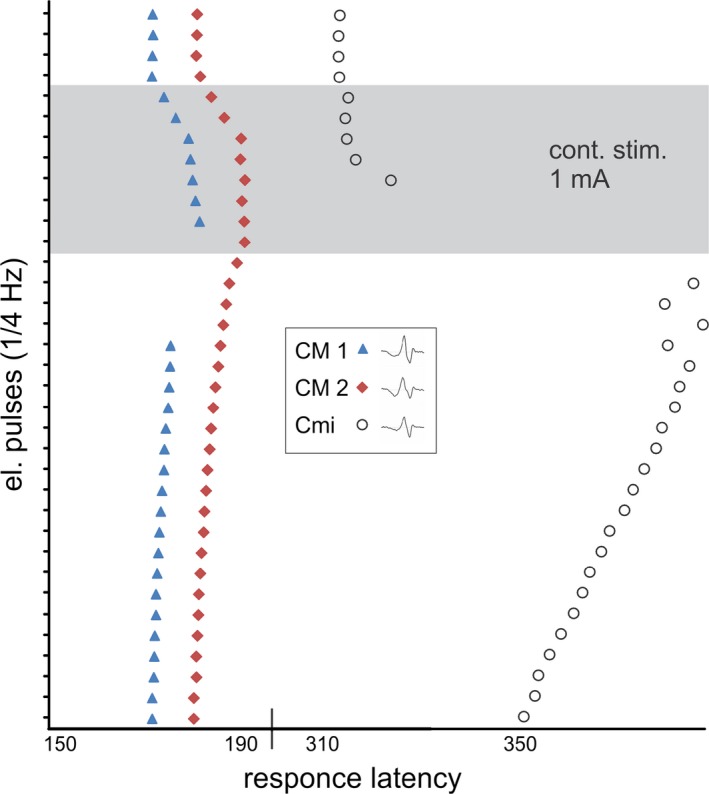
Repetitive electrical pulses (0.5 ms square pulse, 0.25 Hz) given intradermally evoked action potentials from two CM‐nociceptors (CM 1; triangle and CM 2; square) and one CMi‐nociceptor (open circles) depicted in a successive order from top to bottom (falling leaf plot). Additional application of low‐intensity cathodal direct current (1 mA for 30 s: gray frame) was reported by the patient as unpleasant. The two CM‐nociceptors only showed a transient and mild activation as indicated by irregular increases of response latency. In contrast, the CMi‐nociceptor responded vigorously after a delay of about 20 s. This CMi‐nociceptor did not show other obvious signs of hyperexcitability (no spontaneous activity, no mechanical nor heat response) and was negative to phasic electrical stimulation at 30 mA (0.2 ms pulse duration)

In patient 1, normal transcutaneous electrical activation thresholds were found for three CM‐nociceptors and one CMi‐nociceptor (not shown). However, when stimulated transcutaneously by a low‐intensity cathodal direct current (1 mA for 30 s via saline iontophoresis, see Fig. 1), the CMi‐nociceptor showed a vigorous, but delayed, activation starting after ca. 15 s and lasting throughout the 30 s stimulation period. In contrast, during the same stimulation, two CM‐nociceptors showed only a modest response during the first seconds, but later no clear signs of further activation throughout the last part of the stimulation period.

In patient 2, one CMi‐nociceptor had a low electrical threshold at 18 mA (0.2 ms pulse; see (Orstavik et al., 2003) for comparison). This fiber did also show other signs of hyperexcitability such as mechanical sensitization, spontaneous activity as well as a strong activation by heat at 42.5°C which corresponded to the perceived heat pain of the patient.

## 4 Discussion

In these two patients with erythromelalgia‐like pain in the distal extremities and signs of small fiber neuropathy, we found C‐nociceptors with low heat thresholds as well as other signs of changes in nociceptor excitability, including hyperexcitability to electrical stimulation, spontaneous activity, mechanical sensitization, and multiple spikes. Compared to control patients with comparable pain phenotypes, we observed a tendency toward more activity‐dependent slowing (ADS) in the CMi‐nociceptors. Given the role of Nav 1.9 in nociceptor excitability (Dib‐hajj et al., 2010) and recent findings in humans linking Nav 1.9 to both painful neuropathy (Han et al., 2015 *;* Huang et al., 2014), episodic pain (Zhang et al., 2013), cold‐aggravated pain (Leipold et al., 2015 *)* as well as loss of pain perception (Leipold et al., 2013), it is intriguing to hypothesize that a changed function of Nav 1.9 due to the rare genetic variants in these patients might underlie the described findings in C‐nociceptors. However, the results must be interpreted with caution. We have only recorded microneurography data from these two patients (with two different genetic variants) and basic functional electrophysiological characterization of Nav 1.9 has not been performed. Accordingly, whether these variants have functional roles of importance for their phenotype, are still unknown. An approach investigating sensory neurons produced from iPSC (induced pluripotent stem cells) derived from patients, as recently described for Nav 1.7 (Cao et al., 2016), might be useful in this respect.

Although these patients reported high heat pain thresholds during thermal detection testing, the heat activation thresholds of their CM‐ and C‐nociceptors of unknown type tended to be low, both compared to other patients with comparable pain phenotypes and previous recordings from healthy elderly subjects (Namer et al., 2009). The low heat activation thresholds are also in contrast to desensitization observed in parts of the C‐nociceptors in diabetic neuropathy (Orstavik et al., 2006). Although there may be several explanations for lowered heat activation thresholds of nociceptors, one possible mechanism might be depolarization of the sensory endings related to increased persistent sodium currents via Nav 1.9.

CMi‐nociceptors (often denominated silent or sleeping C‐nociceptors) do normally have high electrical activation thresholds, which by far exceed the thresholds of CM‐nociceptors. However, in a recording from patient 1, low‐intensity electrical stimulation revealed a delayed, but pronounced activation of the CMi‐nociceptor, distinctly contrasting a more immediate, but short‐lasting (only for some seconds), response of two CM‐nociceptors. This points to important differences between CMi‐ and CM‐nociceptors, but may also indicate excitability changes related to membrane depolarization, possibly due to increased persistent sodium currents, as demonstrated by Huang et al. (2014) for other Nav 1.9 mutations. In patient 2, a lowered electrical threshold in a CMi‐nociceptor could confirm hyperexcitability, which might be linked to the spontaneous activity and mechanical sensitization observed in this fiber.

A tendency toward more ADS and more pronounced supranormal conduction in the “velocity recovery cycles” in CMi‐nociceptors was observed in these two patients compared to the control patients. Increased ADS has been linked to decreased excitability (De Col, Messlinger, & Carr, 2012), but the relationship between ADS, membrane polarization, and excitability is complex. Both Nav channel inactivation (De Col, Messlinger, & Carr, 2008) and accumulation of intracellular sodium (Tigerholm et al., 2014) are probably involved in ADS, and although depolarization may be expected to decrease ADS (due to Nav channel inactivation), increased sodium influx via Nav 1.9 could hypothetically facilitate accumulation of intracellular sodium and thereby explain higher ADS. Similarly, higher intracellular sodium concentration has also been shown to cause supranormal conduction in the “recovery cycles” in a modeling study (Tigerholm et al., 2015) which could be in line with the presented results of CMi‐nociceptors in our patients. Higher intracellular sodium concentrations might also cause injury to small nerve fibers (Persson et al., 2010) and explain a subsequent small fiber neuropathy which could result in a loss of spatial summation. Less spatial summation together with hyperexcitability of remaining fibers may serve as one possible explanation for the apparent mismatch between the relatively high heat pain thresholds perceived and the low heat activation thresholds in subgroups of heat‐sensitive C‐nociceptors.

## Funding Information

German Research Council (DFG Na 970 1/1, SFB 1158).

## Conflict of Interest

None declared.
